# Audit on venous thromboembolism risk assessment in mental health inpatient wards

**DOI:** 10.1192/bjo.2021.870

**Published:** 2021-06-18

**Authors:** Rahul Malhotra

**Affiliations:** Bryn Y Neuadd Hospital, Betsi Cadwaladr University Health Board

## Abstract

**Aims:**

From May 2015 NHS organisations in Wales are expected to report the number of VTE cases associated with hospital admissions which are possible hospital acquired thrombosis (HAT) per calendar month. NICE Quality Standards (QS3) recommend that All patients, on admission, receive an assessment of VTE and bleeding risk using the clinical risk assessment criteria described in the national tool.

**Background:**

VTE is a condition in which a blood clot (thrombus) forms in a vein, most commonly in the deep veins of the legs, known as a deep vein thrombosis (DVT). The thrombus can dislodge from its original site and travel in the blood (embolism). If it becomes lodged in the lungs, a condition known as a pulmonary embolism (PE) arises and can cause sudden death. Hospital acquired thrombosis is avoidable and unfortunately kills patients under our care.

**Method:**

Collected data using a standardised form for 131 patients from 3 inpatient mental health units on documentation of a VTE risk assessment in the inpatient notes. For those patients who had a documented risk assessment, further data were collected on documentation of contraindications, eisk factors, sign and date of prescription and the appropriateness of prescribing.

**Conclusion:**

8% of patients from one mental health unit (n = 48) had a documented risk assessment in the notes. The subsections of documented risk assessment including contraindications, risk factors, sign and date of prescriptions and appropriateness of prescribing were complete at 100%. No patients from the other 2 mental health units (n = 39,44) had a risk assessment documented in the notes.

Recommendations: All adult inpatients in Mental Health units must receive a venous thrombo-embolism risk assessment. This must be documented on the Inpatient Medication Chart. Consider adding a risk assessment checklist tool mapped from the Department of Health guidelines into the Mental Health Inpatient Clerking in pro-forma.

